# Antioxidant Ascorbic Acid Modulates NLRP3 Inflammasome in LPS-G Treated Oral Stem Cells through NFκB/Caspase-1/IL-1β Pathway

**DOI:** 10.3390/antiox10050797

**Published:** 2021-05-18

**Authors:** Jacopo Pizzicannella, Luigia Fonticoli, Simone Guarnieri, Guya D. Marconi, Thangavelu Soundara Rajan, Oriana Trubiani, Francesca Diomede

**Affiliations:** 1 “Ss. Annunziata” Hospital, ASL 02 Lanciano-Vasto-Chieti, 66100 Chieti, Italy; jacopo.pizzicannella@unich.it; 2 Department of Innovative Technologies in Medicine & Dentistry, University “G. d’Annunzio” Chieti-Pescara, via dei Vestini, 31, 66100 Chieti, Italy; luigia.fonticoli@unich.it (L.F.); oriana.trubiani@unich.it (O.T.); 3 Department of Neuroscience, Imaging and Clinical Sciences, Center for Advanced Studies and Technology (CAST), University “G. d’Annunzio” Chieti-Pescara, via dei Vestini, 31, 66100 Chieti, Italy; simone.guarnieri@unich.it; 4 Department of Medical, Oral and Biotechnological Sciences, University “G. d’Annunzio” Chieti-Pescara, via dei Vestini, 31, 66100 Chieti, Italy; guya.marconi@unich.it; 5 Department of Biotechnology, Karpagam Academy of Higher Education, Coimbatore 641 021, India; tsrajanpillai@gmail.com

**Keywords:** periodontal disease, NLRP3 inflammasome, ascorbic acid, gingival mesenchymal stem cells, *Porphyromonas gingivalis*

## Abstract

Human gingival mesenchymal stem cells (hGMSCs) and endothelial committed hGMSCs (e-hGMSCs) have considerable potential to serve as an in vitro model to replicate the inflammation sustained by *Porphyromonas gingivalis* in periodontal and cardiovascular diseases. The present study aimed to investigate the effect of ascorbic acid (AA) on the inflammatory reverting action of lipopolysaccharide (LPS-G) on the cell metabolic activity, inflammation pathway and reactive oxygen species (ROS) generation in hGMSCs and e-hGMSCs. Cells were treated with LPS-G (5 μg mL^−1^) or AA (50 μg mL^−1^) and analyzed by 3-(4,5-Dimethylthiazol-2-yl)-2,5-Diphenyltetrazolium Bromide (MTT) assay, immunofluorescence and Western blot methods. The rate of cell metabolic activity was decreased significantly in LPS-G-treated groups, while groups co-treated with LPS-G and AA showed a logarithmic cell metabolic activity rate similar to untreated cells. AA treatment attenuated the inflammatory effect of LPS-G by reducing the expression of TLR4/MyD88/NFκB/NLRP3/Caspase-1/IL-1β, as demonstrated by Western blot analysis and immunofluorescence acquisition. LPS-G-induced cells displayed an increase in ROS production, while AA co-treated cells showed a protective effect. In summary, our work suggests that AA attenuated LPS-G-mediated inflammation and ROS generation in hGMSCs and e-hGMSCs via suppressing the NFκB/Caspase-1/IL-1β pathway. These findings indicate that AA may be considered as a potential factor involved in the modulation of the inflammatory pathway triggered by LPS-G in an vitro cellular model.

## 1. Introduction

Ascorbic acid (AA) is well known to perform a key role in the maintenance of tissue integrity, scavenging free radicals, and has demonstrated immunomodulatory properties during chronic inflammatory diseases. In periodontitis, AA showed a deceleration in the progression of tissue loss and induced regeneration by stimulating the progenitor cells of the periodontal ligament commitment [1]. Periodontal disease (PD) can be defined as a complex oral disease and its treatment represents a challenging condition for clinicians [2]. Periodontal disease involves chronic inflammation sustained by various types of bacteria that accumulate in dental plaque and cause localized inflammation by producing various pro-inflammatory factors, which include C-reactive protein (CRP), interleukin (IL)-1β, IL-6, tumor necrosis factor (TNF)-α and matrix metalloproteinases (MMP) [3]. The progression of periodontitis leads to the destruction of deep periodontal pockets and causes systemic disease processes upon the increased expression of pro-inflammatory factors. Indeed, periodontitis represents a possible risk factor for several systemic diseases, including cardiovascular disease (CVD) [4].

There are a wide range of disorders that affect the heart and blood vessels with complex pathogenic mechanisms. The pathogenesis is based on the presence of a high level of low-density lipoprotein cholesterol in the blood, which affects the cellular permeability and modulates the integrity of arterial walls. A strong link has been found between periodontitis and cardiovascular diseases as both diseases show common inflammatory pathways, share similar risk factors and start with tissue damage [5,6,7].

The presence of Gram-negative anaerobic pathogens in the periodontal pockets is critical for the invasion of the deeper tissues, reaching the blood circulation and inducing a systemic immune response away from the original niche [8].

Several studies reported that periodontal pathogens are associated with chronic inflammation and lead to epithelial barrier dysfunction, causing a loss of epithelial sheet integrity and producing microulceration [9,10,11]. *Porphyromonas gingivalis* (*P. gingivalis*) is one of the bacteria involved in the biofilm development of bacterial plaque and plays a vital part in the advancement of periodontal illness. Lipopolysaccharide (LPS-G) is the virulence factor of *P. gingivalisis*, which induces the host immune response via activating the innate and acquired immunity [12].

To study the in vitro response to LPS-G and inflammatory pathway modulation, different mesenchymal stem cell (MSC) populations have been used [13,14]. Recently, human gingival mesenchymal stem cells (hGMSCs) have attracted the attention of many researchers. They met the minimal criteria proposed by Dominici et al. [15] to characterize MSCs. hGMSCs showed self-renewal capacity, mesengenic differentiation ability under induction conditions and the expression of MSC markers [16].

The inflammasome NLRP3 is activated during periodontal disease, which is most often associated with TLR4 activation. NLRP3 initiates the inflammatory cascade by activating the release of interleukin (IL)-1β that regulates the degree and progression of the inflammation.

Ascorbic acid is a water-soluble molecule required for human health. The human body is unable to synthesize vitamin C endogenously and thus requires external sources of vitamin C from dietary intake and supplementation [17]. AA is an effective antioxidant that relieves oxidative stress and participates in a variety of biochemical reactions. Moreover, AA is able to decrease the responses of some inflammatory biomarkers and several pro-inflammatory cytokines [18,19,20].

The role of AA in the suppression of the activation of inflammasome cascade TLR4/NFκB signaling that leads to the release of IL-1β in periodontitis is yet to be determined. Accordingly, the aim of our research was to explore the role of AA in LPS-G-stimulated hGMSCs and endothelial differentiated hGMSCs (e-hGMSCs) to evaluate the regulation of the inflammatory cascade.

## 2. Materials and Methods

### 2.1. Ethic Statement

The present research project was approved by the Medical Ethics Committee at the Medical School, “G. d’Annunzio” University, Chieti, Italy (n°266/14). All enrolled subjects signed an informative consent form.

### 2.2. Cell Culture

Cells were isolated from human gingival tissue biopsies from two different donors as previously described [21]. Biopsies were washed with phosphate-buffered saline (PBS) (Lonza, Basel, Switzerland), cut into small pieces and then placed in a TheraPEAK™MSCGM-CD™ Bullet Kit serum free, chemically defined medium for the growth of human mesenchymal stem cells (MSCGM-CD, Lonza, Basel, Switzerland) at 37 °C in a controlled atmosphere (5% CO_2_). The medium was refreshed twice a week. After 2 weeks of culture, cells were spontaneously migrated from the tissue explants. After reaching around 80% of confluence, cells were trypsinized using trypsin-EDTA (Lonza, Basel, Switzerland) and subcultured until passage 2 (P2). All the following experiments were conducted in triplicate and were repeated 3 times.

### 2.3. Cell Characterization

Human Gingival Mesenchymal Stem Cells (GMSC)s were characterized by cytofluorimetric and morphological analysis and the capacity to differentiate into osteogenic and adipogenic lineage. Fluorescence-Activated Cell Sorting (FACS) analysis showed the expression of CD14, CD34, CD45, CD73, CD90 and CD105 as previously reported [22]. Briefly, cells were stained for CD45, CD73 and CD90 with fluorescein isothiocyanate-conjugated anti-human antibodies and for CD14, CD34 and CD105 with phycoerythrin-conjugated antibodies. After staining procedures, a FACStar-plus flow-cytometry system running Cell-Quest software (version 5.2.1, Becton-Dickinson, Mountain View, CA, USA) was used. All reagents used for flow cytometry were purchased from Becton Dickinson (Milan, Italy). To study the morphological features, hGMSCs were fixed with paraformaldehyde, stained with toluidine blue and observed with a Leica microsystem microscope. To evaluate the capacity to differentiate into osteogenic and adipogenic lineages, hGMSCs were incubated with MSCGM-CD (Lonza, Basel, Switzerland) medium with osteogenic supplements and in adipogenesis induction/maintenance medium (Lonza, Basel, Switzerland), respectively. Osteogenic differentiation was evaluated using alizarin red S staining (Sigma-Aldrich, Milan, Italy) and the adipogenic commitment was stained with oil red O solution (Lonza Basel, Switzerland). To validate the colorimetric detection, a real-time polymerase chain reaction (RT-PCR) was performed, evaluating the expression of Runt-related transcription factor-2 (RUNX-2), alkaline phosphatase (ALP), fatty acid-binding protein 4 (FABP4) and peroxisome proliferator-activated receptor γ (PPARγ) after 28 days of differentiation. Commercially available TaqMan Gene Expression Assays (RUNX-2Hs00231692_m1; ALP Hs01029144_m1; FABP4 Hs01086177_m1;PPARγ Hs01115513_m1) and the TaqManUniversal PCR Master Mix (Applied Biosystems, Foster City, CA, USA) were used according to standard protocols. Beta-2 microglobulin (B2MHs99999907_m1) (Applied Biosystems, Foster City, CA, USA) was used for template normalization [23].

### 2.4. Endothelial Differentiation

Endothelial differentiation procedures to obtain the e-hGMSCs were started with 50–60% of cell confluency. Endothelial Growth Medium (EGM-2, Lonza, Basel, Switzerland), composed of EGM-2 Bullet Kit (Lonza, Basel, Switzerland) growth supplements containing hydrocortisone, human Fibroblast Growth Factor (hFGF-b), R3-Insulin-like Growth Factor-1 (R3-IGF-1), ascorbic acid, human Epithelial Growth Factor (hEGF), GA-1000, heparin, 5% FBS and 50 ng/mL of Vascular Endothelial Growth Factor-165 (VEGF-165), was used. Cells were maintained at 37 °C with 5% CO_2_ for 14 days [24].

### 2.5. Tube Formation Test

In this study, 12-well culture plates pretreated with Cultrex^®^ Basement Membrane Extract (Trevigen Inc., Gaithersburg, MD, USA) (300 μL/well) were used in the tube formation assay. Human GMSCs and e-hGMSCs were seeded at a density of 2 × 10^5^ cells per well after matrix solidification (at 37 °C for 30 min). Capillary-like tube structures have been observed using an inverted light microscope at phase contrast after 4 h of culture to 24 h [24].

### 2.6. Study Design

All experiments were performed in triplicate with hGMSCs at P2.

The study was organized with the following groups:

- Untreated hGMSCs, used as negative control (CTRL);
- hGMSCs treated for 24 h with 50 μg mL^−1^ AA (AA);
- hGMSCs treated for 24 h with 5 μg mL^−1^ ultrapure LPS-G from *P. gingivalis* (tlrl-ppglps, InvivoGen, San Diego, CA, USA) (LPS-G);
- hGMSCs co-treated for 24 h with 50 μg mL^−1^ AA and 5 μgmL^−1^ LPS-G (AA + LPS-G);
- Untreated e-GMSCs, used as negative control (e-CTRL);
- e-hGMSCs treated for 24 h with 50 μg mL^−1^ AA (e-AA);
- e-hGMSCs treated for 24 h with 5 μg mL^−1^ ultrapure LPS-G (tlrl-ppglps, InvivoGen, San Diego, CA, USA; e-LPS-G);
- e-hGMSCs co-treated for 24 h with 50 μgmL^−1^ AA and 5 μg mL^−1^ LPS-G (AA + e-LPS-G).

Based on our previously obtained data (data not shown), we found, after an LPS-G titration tested on oral stem cells, that 5 μg mL^−1^ LPS-G was the optimal concentration to be utilized in the current study [25].

### 2.7. Cell Metabolic Activity

3-(4,5-Dimethylthiazol-2-yl)-2,5-Diphenyltetrazolium Bromide (MTT) colorimetric assay was used for all experimental groups to determine the cell viability as previously reported [26]. Human GMSCs were seeded with a density of 2 × 10^3^ cells/well in a 96-well plate. At 24, 48 and 72 h of culture, 20 μL of MTT (CellTiter 96 AQueous One Solution reagent, Promega, Milan, Italy) solution was added to each well. Plates were maintained in the incubator for 3 h and then were read at 490 nm wavelength using a microplate reader (Synergy HT, BioTek Instruments, Winooski, VT, USA).

### 2.8. Molecular Pathway

Samples were fixed with 4% paraformaldehyde in 0.1 M of PBS (Lonza, Basel, Switzerland). Then, cells were permeabilized with 0.5% Triton X-100 in PBS (Lonza, Basel, Switzerland) for 10 min and blocked with 5% skimmed milk in PBS for 30 min [27]. The following primary antibodies were used in the study: anti-TLR4 (1:200; Santa Cruz Biotechnology, Dallas, TX, USA), anti-MyD88 (1:200; Santa Cruz Biotechnology, Dallas, TX, USA), anti-NFκB (1:200; Santa Cruz Biotechnology, Dallas, TX, USA), anti-NLRP3(1:500; Novus, Centennial, CO, USA), anti-Caspase-1 (1:200; Santa Cruz Biotechnology, Dallas, TX, USA) and anti-IL-1β (5 µg/mL; ThermoFisher, Waltham, MA, USA). Cells were incubated with primary antibody for 2 h at room temperature. Then, samples were incubated with Alexa Fluor 568 red fluorescence conjugated goat anti-rabbit secondary antibody (1:200; Molecular Probes, Invitrogen, Eugene, OR, USA) for 1 h at 37 °C. To stain the cytoskeleton actin, cells were treated with Alexa Fluor 488 phalloidin green fluorescent conjugate (1:400, Molecular Probes, Invitrogen, Eugene, OR, USA) for 1 h, and to stain the nuclei, cells were stained with TOPRO (1:200; Molecular Probes, Invitrogen, Eugene, OR, USA) for 1 h. The Zeiss LSM800 confocal system (Zeiss, Jena, Germany) was used to acquire microphotographs.

### 2.9. Western Blot Analysis

Proteins (50 μg) from all samples were processed as previously described [28]. Membranes were incubated for 12 h at 4 °C with primary antibodies to anti-TLR4 (1:500; Santa Cruz Biotechnology, Dallas, TX, USA), anti-MyD88 (1:500; Santa Cruz Biotechnology, Dallas, TX, USA), anti-NFκB (1:500; Santa Cruz Biotechnology, Dallas, TX, USA), anti-NLRP3 (3 µg/mL; Novus, Dallas, TX, USA), anti-Caspase-1(1:500; Santa Cruz Biotechnology, Dallas, TX, USA), anti-IL-1β (1 µg/mL; Thermo Fisher, Waltham, MA, USA) and β-actin (1:1000; Santa Cruz Biotechnology, Dallas, TX, USA). Samples were maintained at room temperature for 30 min with peroxidase-conjugated secondary antibody diluted 1:1000 in 1 × Tris Buffered Saline (TBS), 5% milk and 0.05% Tween-20 (Signa-Aldrich, Milan, Italy) [29]. To visualize protein bands, the Electrochemiluminescence (ECL) method was used, and the protein levels were measured by means of the Bio-Rad Protein Assay (Bio-Rad Laboratories, Hercules, CA, USA).

### 2.10. Reactive Oxygen Species (ROS) Evaluation

Human and endothelial differentiated GMSCs were seeded in a 35 mm imaging dish (µ-Dish, ibidi GmbH, Gräfelfing, Germany) and treated for 24 h in culture medium containing 5 µg mL^−1^ LPS-G (hGMSCs + LPS-G or e-hGMSCs + LPS-G) or 5 µg mL^−1^ LPS-G plus 50µg/mL ascorbic acid (hGMSCs + AA/LPS-G or e-hGMSCs + AA/LPS-G) or 50µg/mL ascorbic acid (hGMSCs + AA or e-hGMSCs+ AA) or culture medium alone (hGMSCs, or e-hGMSCs). At the end of the expected time, incubation medium was removed and the cells were washed with Normal External Solution (NES) containing (in mM) 125 NaCl, 5 KCl, 1 MgSO4, 1 KH_2_PO_4_, 5.5 glucose, 1 CaCl_2_, 20 4-(2-hydroxyethyl)-1-piperazineethanesulfonic acid (HEPES), pH 7.4 and incubated with 10 μM of 2′,7′-dichlorodihydrofluorescein diacetate (H2DCFDA, Thermo Fisher, Waltham, MA, USA) at 37 °C in a humidified incubator (for 30 min) maintaining for all procedures the respective culture media treatments. At the end of dye incubation, the cells were washed with NES and observed in NES alone (hGMSCs or e-hGMSCs) or maintained in NES plus LPS-G, LPS-G and AA or AA alone as expected. For each condition, confocal images were randomly acquired by means of motorized table SMC 2009 and multiple single position acquisition function (Tiles-Advanced setup, carrier 35 mm petri dish) of Zen Blue software (Zen 3.0 SR, Carl Zeiss, Jena, Germany) using a Zeiss LSM800 microscope (Carl Zeiss, Jena, Germany), equipped with an inverted microscope Axio-obserber D1 (Carl Zeiss, Jena, Germany) and an objective W-Plan-Apo 40 X/1.3 DIC (Carl Zeiss, Jena, Germany). Excitation was fixed at 488 nm and emission collected with the filter set over 505–530 nm. The acquisition settings were kept constant between specimens. Offline image analyses were performed using Fiji distribution of ImageJ (version 1.53c, National Institutes of Health, Bethesda, MD, USA) measuring for each acquired cell the mean of fluorescence intensity (arbitrary units, F) and the area of the measured cells (µm^2^). Quantitative data of ROS production are expressed as ratio F/µm^2^.

### 2.11. Statistical Analysis

Statistical evaluation was performed using GraphPad Prism 4.0 software (GraphPad, San Diego, CA, USA) using *t*-test and ordinary one-way ANOVA followed by post hoc Bonferroni’s multiple comparisons tests. Values of *p* < 0.01 were considered statistically significant.

## 3. Results

### 3.1. Immunophenotype and In Vitro Differentiation Ability of Isolated hGMSCs

**Figure 1:**
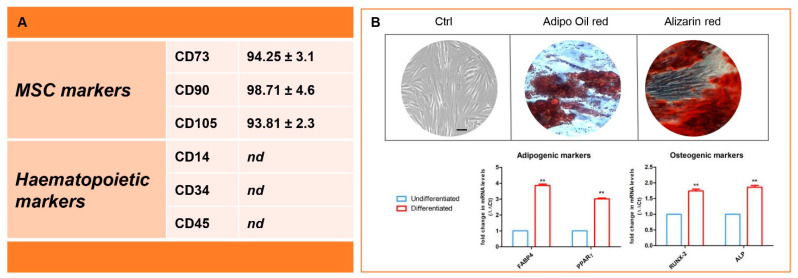
Human Gingival Mesenchymal Stem Cells (hGMSCs) characterization. (**A**) Antigen expression quantification by flow cytometry in human Gingival Mesenchymal Stem Cells (hGMSCs). Cells do not express hematopoietic antigens CD14, CD34 and CD45 and co-express CD73, CD90 and CD105. (**B**) Differentiation assay: cultured hGMSCs were induced to differentiate into adipocytes and osteoblast. Adipogenic differentiation was demonstrated by lipid vacuole detection with the lipid adipo oil red staining; and calcium deposits in the osteoblast differentiation were detected with alizarin red staining. Graph bars showed the expression of specific markers in cells undergoing adipogenic and osteogenic differentiation. Results represent three independent experiments performed in triplicate (*n* = 3). MSC: mesenchymal stem cell; nd: not detectable; FABP4: fatty acid-binding protein 4; PPARγ: peroxisome proliferator-activated receptor γ; RUNX-2: Runt-related transcription factor-2; ALP: alkaline phosphatase. Scale bar: 20 µm. ** *p* < 0.01.

Characterization analysis showed that hGMSCs were positive for CD73, CD90 and CD105, respectively. Confirming their mesenchymal phenotype, cells were negative for hematopoietic markers (Figure 1A). Data presented are mean ± SD (*n* = 3). To assay differentiation capacity to adipocytes and osteoblasts, hGMSCs were induced with specific differentiation kit media (Lonza, Basel, Switzerland). Cells treated with adipogenic medium produced numerous vacuoles detected with adipo oil red solution (Figure 1B, central panel). Furthermore, after treating hGMSCs with the osteogenic differentiation medium, the formation of calcium deposits revealed by alizarin red staining was observable (Figure 1B, right panel). These results indicate that hGMSCs can differentiate into both lineages. To validate the differentiation ability, RT-PCR was performed to evaluate the expression for adipogenic- and osteogenic-specific markers. In differentiated cells, FABP4, PPARγ, RUNX2 and ALP were upregulated when compared to the control cells (Figure 1B, down panel).

### 3.2. Endothelial Differentiation and Tube Formation of hGMSCs

**Figure 2:**
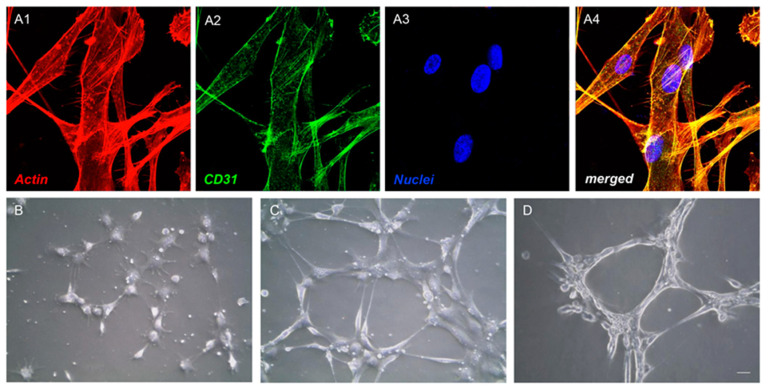
Endothelial differentiation and tube formation of hGMSCs. (**A1**–**A4**) After 14 days of exposure to endothelial differentiation, endothelial differentiated- human Gingival Mesenchymal Stem Cells (e-hGMSCs) showed positivity for endothelial marker, CD31. (**B**–**D**) After culture under endothelial differentiation conditions for 24 h, hGMSCs were seeded onto Cultrex to allow the formation of capillary-like structures. Cells were observed by means light microscopy after 2 (h) (**B**), 4 h (**C**) and 6 h (**D**) after seeding on dishes pre-treated with Cultrex. (**A1**–**A4**): magnification, 40×; (**B**–**D**): scale bar, 20 µm.

An evaluation of the impact of the differentiation medium kit exposure on hGMSCs to induce the endothelial transition (e-hGMSCs) was performed by examining the typical endothelial markers using CD31 immunofluorescence via staining. As reported, differentiated cells showed the expression of CD31 observed under confocal laser scanning microscopy (Figure 2A1–A4). Results of the tube formation assay showed that a number of capillary-like structures were observed after 24 h of differentiation induction (Figure 2B–D).

### 3.3. LPS-G Affect the Cell Metabolic Activity of hGMSCs

**Figure 3:**
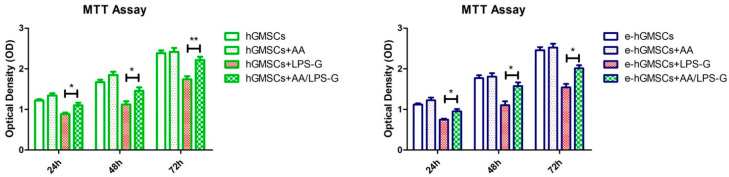
Effects of Lipopolysaccharide from *Porphyromonas gingivalis* (LPS-G) and ascorbic acid (AA) on the cell metabolic activity of hGMSCs and e-hGMSCs. Cell growth curve of hGMSCs and e-hGMSCs detected by absorbance at 490 nm showed the different cell metabolic activity of cells under treatment with AA, LPS-G and LPS-G + AA. * *p* > 0.05; ** *p* < 0.01.

At defined time points, 24, 48 and 72 h after cultivation in all considered culture conditions hGMSCs, the cell metabolic activity was evaluated. Human GMSC and e-hGMSC cell metabolic activity was reduced when treated with LPS-G compared to the untreated cells. Conversely, constant cell numbers were recorded in the untreated and AA-treated groups. In addition, cells co-treated with AA and LPS-G showed a better trend in cell metabolic activity at all considered end points (Figure 3).

### 3.4. The TLR4/MyD88/NFκB/NLRP3/Caspase-1/IL-1β Signaling Pathway Was Involved in AA Anti-inflammatory Effects on LPS-G-Stimulated Cells

**Figure 4:**
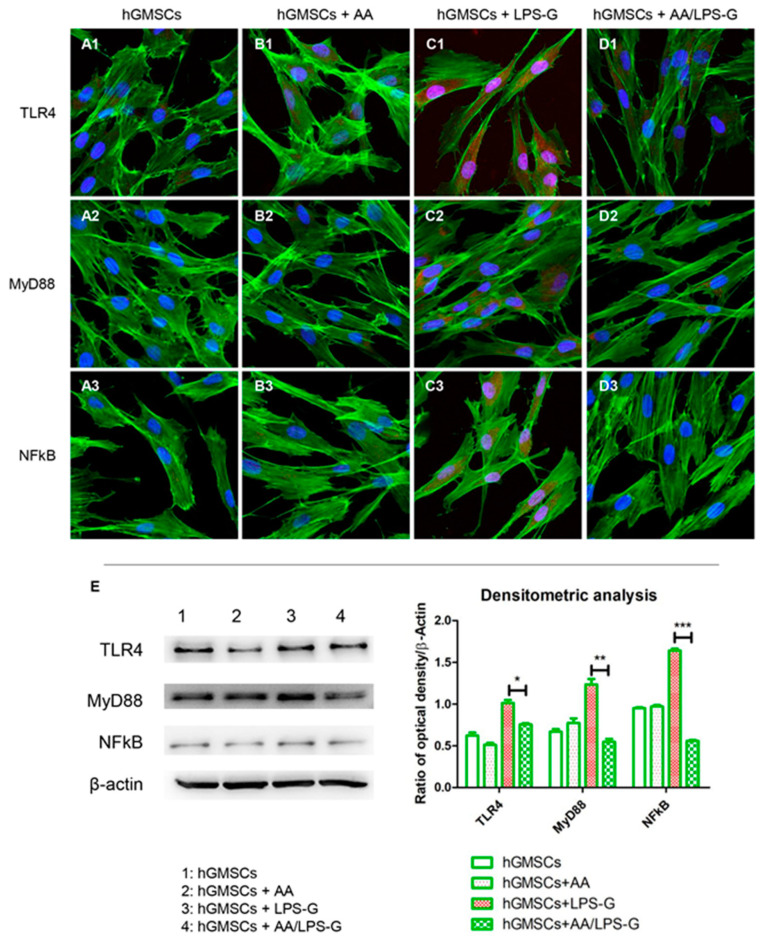
TLR4/MyD88/NFκB signaling pathway and differences in protein levels in hGMSCs. Expression of TLR4, MYD88 and NFκB, examined by confocal microscopy and Western blot experiments. (**A1**–**D1**) TLR4 expression in hGMSCs (CTRL), hGMSCs + AA, hGMSCs + LPS-G, hGMSCs + AA/LPS-G. (**A2**–**D2**) MyD88 expression in hGMSCs (CTRL), hGMSCs + AA, hGMSCs + LPS-G, hGMSCs + AA/LPS-G. (**A3**–**D3**) NFκB expression in hGMSCs (CTRL), hGMSCs + AA, hGMSCs + LPS-G, hGMSCs + AA/LPS-G. (**E**) TLR4, MyD88 and NFκB protein levels. β-actin was used as a loading control. Green fluorescence: cytoskeleton actin. Red fluorescence: TLR4, MYD88, NFκB. Blue fluorescence: cell nuclei. Scale bar: 20 µm. * *p* < 0.05; ** *p* < 0.01; *** *p* < 0.001.

**Figure 5:**
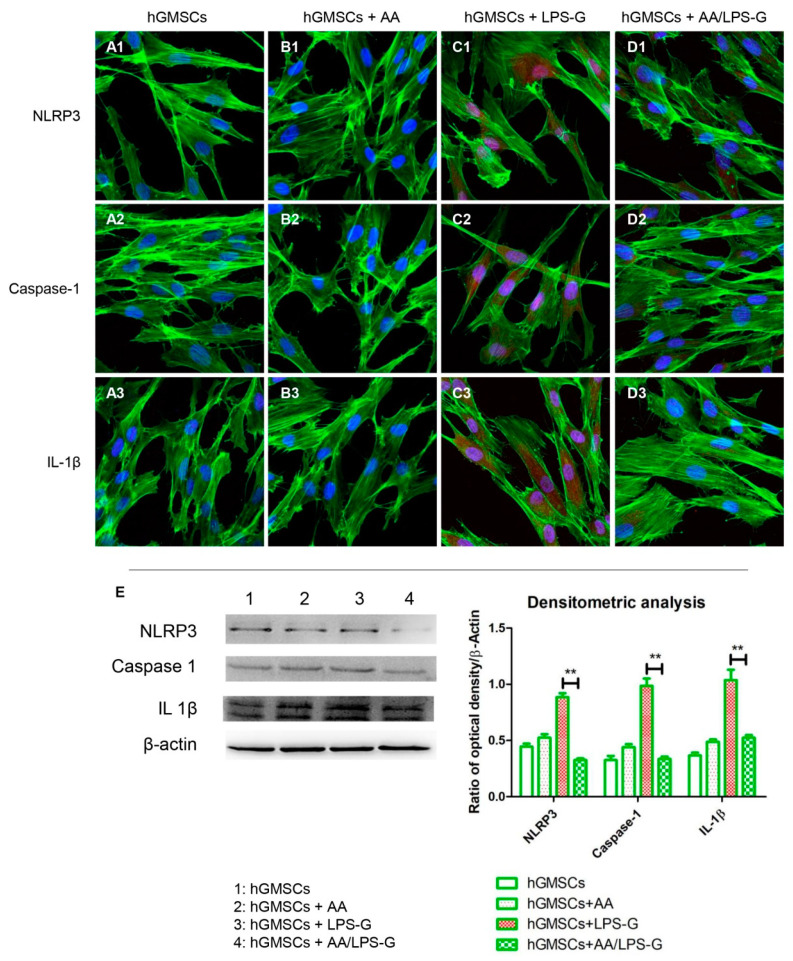
NLRP3/Caspase-1/IL-1β signaling pathway and differences in protein levels in hGMSCs. Expression of NLRP3, Caspase-1 and IL-1β, examined by confocal microscopy and Western blot experiments. (**A1**–**D1**) NLRP3 expression in hGMSCs (CTRL), hGMSCs + AA, hGMSCs + LPS-G, hGMSCs + AA/LPS-G. (**A2**–**D2**) Caspase-1 expression in hGMSCs (CTRL), hGMSCs + AA, hGMSCs + LPS-G, hGMSCs + AA/LPS-G. (**A3**–**D3**) IL-1β expression in hGMSCs (CTRL), hGMSCs + AA, hGMSCs + LPS-G, hGMSCs + AA/LPS-G. (**E**) NLRP3, Caspase -1 and IL-1β protein levels. β-actin was used as a loading control. Green fluorescence: cytoskeleton actin. Red fluorescence: NLRP3, Caspase-1, IL-1β. Blue fluorescence: cell nuclei. Scale bar: 20 µm. ** *p* < 0.01.

**Figure 6:**
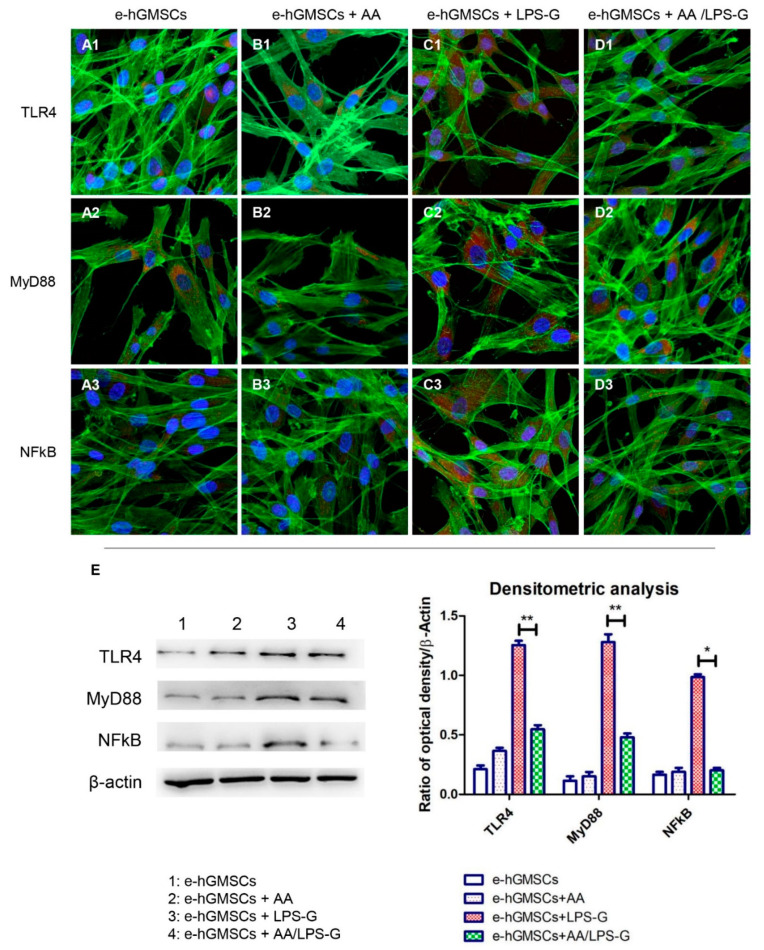
TLR4/MyD88/NFκB signaling pathway and differences in protein levels in e-hGMSCs. Expression of TLR4, MYD88 and NFκB, examined by confocal microscopy and Western blot experiments. (**A1**–**D1**) TLR4 expression in e-hGMSCs (CTRL), e-hGMSCs + AA, e-hGMSCs + LPS-G, e-hGMSCs + AA/LPS-G. (**A2**–**D2**) MyD88 expression in e-hGMSCs (CTRL), e-hGMSCs + AA, e-hGMSCs + LPS-G, e-hGMSCs + AA/LPS-G. (**A3**–**D3**) NFκB expression in e-hGMSCs (CTRL), e-hGMSCs + AA, e-hGMSCs + LPS-G, e-hGMSCs + AA/LPS-G. (**E**) TLR4, MyD88 and NFκB protein levels. β-actin was used as a loading control. Green fluorescence: cytoskeleton actin. Red fluorescence: TLR4, MYD88, NFκB. Blue fluorescence: cell nuclei. Scale bar: 20 µm. * *p* < 0.05; ** *p* < 0.01.

**Figure 7:**
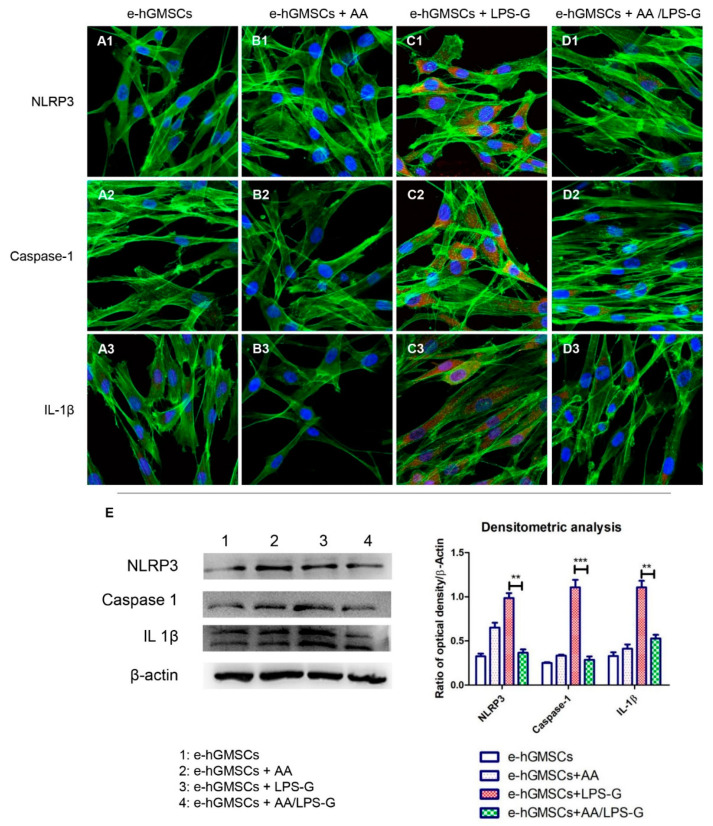
NLRP3/Caspase-1/IL-1β signaling pathway and differences in protein levels in e-hGMSCs. Expression of NLRP3, Caspase-1 and IL-1β, examined by confocal microscopy and Western blot experiments. (**A1**–**D1**) NLRP3 expression in e-hGMSCs (CTRL), e-hGMSCs + AA, e-hGMSCs + LPS-G, e-hGMSCs + AA/LPS-G. (**A2**–**D2**) Caspase-1 expression in e-hGMSCs (CTRL), e-hGMSCs + AA, e-hGMSCs + LPS-G, e-hGMSCs + AA/LPS-G. (**A3**–**D3**) IL-1β expression in e-hGMSCs (CTRL), e-hGMSCs + AA, e-hGMSCs + LPS-G, e-hGMSCs + AA/LPS-G. (**E**) NLRP3, Caspase-1 and IL-1β protein levels. β-actin was used as a loading control. Green fluorescence: cytoskeleton actin. Red fluorescence: NLRP3, Caspase-1, IL-1β. Blue fluorescence: cell nuclei. Scale bar: 20 µm. ** *p* < 0.01; *** *p* < 0.001.

Stimulation of hGMSCs and e-hGMSCs with LPS-G induced the expression of the inflammatory pathway. Cells showed an overexpression of TLR4, MyD88, NF-κB, NLRP3, Caspase-1 and IL-1β as observed under confocal microscopy (Figure 4C1–C3, Figure 5C1–C3, Figure 6C1–C3, Figure 7C1–C3). Untreated hGMSCs and e-hGMSCs (CTRL) and AA-treated cells showed lower positivity for TLR4, MyD88, NFκB, NLRP3, Caspase-1 and IL-1β compared to LPS-G-stimulated cells (Figure 4A1–B3, Figure 5A1–B3, Figure 6A1–B3, Figure 7A1–B3). The co-treatment of AA and LPS-G in all experimental groups (hGMSCS and e-hGMSCs) showed a reduction in the expression of studied proteins, indicating a possible protective effect exerted by AA (Figure 4D1–D3, Figure 5D1–D3, Figure 6D1–D3, Figure 7D1–D3). This finding was further confirmed by Western blot analyses, which showed that the expression of TLR4, MyD88, NFκB, NLRP3, Caspase-1 and IL-1β was increased in LPS-G-treated samples. The cells co-treated with AA and LPS-G showed a reduction in the expression of proteins involved in the inflammatory pathway (Figure 4E, Figure 5E, Figure 6E and Figure 7E).

### 3.5. ROS Production

**Figure 8:**
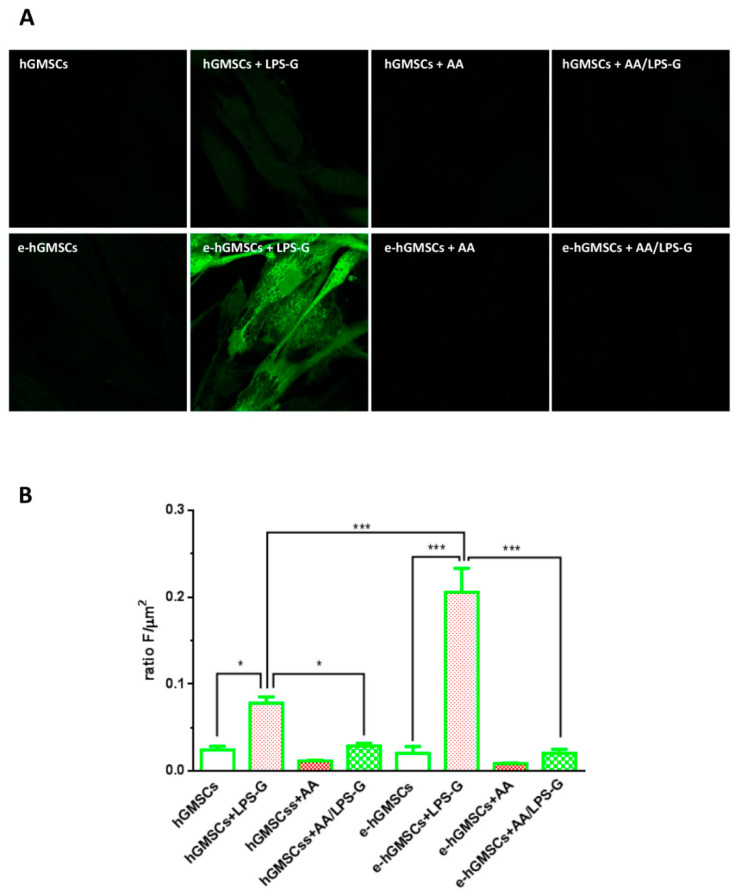
ROS measurements. (**A**) Representative images of H2DCFDA-loaded cells. (**B**) Analysis of ROS production calculated as arbitrary unit of fluorescence per cell surface unit (F/µm2). Data are expressed as mean± S.E.M (hGMSCs *n* = 166, hGMSCs + LPS-G *n* = 148, hGMSCs + AA *n* = 100; hGMSCs + AA/LPS-G *n* = 176; e-hGMSCs *n* = 85, e-hGMSCs + LPS-G *n* = 195, e-hGMSCs + AA *n* = 159; e-hGMSCs + AA/LPS-G *n* = 125, for each experimental condition *n* = 3). * *p* < 0.05; *** *p* < 0.001.). Statistical analysis was performed by one-way ANOVA and post hoc Bonferroni. Scale bar: 20 µm.

ROS production induced by LPS-G has been studied in hGMSCs and e-hGMSCs loaded with the cell-permeant H2DCFDA. Once penetrated inside the cell, the cleavage of the acetate groups by intracellular esterases makes the molecule active and able to undergo oxidation. In this way, the nonfluorescent H2DCFDA is converted to the highly fluorescent 2′,7′-dichlorofluorescein (DCF) by ROS. Images were acquired in live cells by means of confocal microscopy as reported in Figure 8A and the single cells’ fluorescence recorded was analyzed offline. Quantitative results (Figure 8B) showed a significative increase in ROS production both in 5 µg mL^−1^ LPS-G-treated hGMSCs and e-hGMSCs vs. the control condition (hGMSCs and e-hGMSCs, respectively). Interestingly, the co-incubation of LPS-G together with AA counteracted the LPS-G increase in ROS production. Of note, comparing the results obtained in the hGMSCs and e-hGMSCs LPS-G-treated cells, we observed that the latter were more prone to produce ROS.

## 4. Discussion

Periodontitis is a form of chronic inflammatory disease that invades teeth-supporting tissues, sustained mostly by Gram-negative microbes such as *Porphyromonas gingivalis* through lipopolysaccharide (LPS-G) action, which represents one of the major virulence factors in periodontitis progression [30]. Outer membrane endotoxins can trigger multiple signaling pathways that are involved several intracellular events, which eventually stimulate the production of many inflammatory factors and cause the destruction of the periodontal soft tissue, the resorption of alveolar bone and, finally, tooth loss [31].

The inflammasome activation is strictly related to the development and progression of periodontal disease. The establishment of an in vitro model of periodontal inflammation is necessary to study periodontitis’ pathogenesis and the potential novel treatments for this complex disease [32].

The in vitro model established by Pizzicannella et al. [24] attempts to reproduce the microenvironment of periodontal disease using hGMSCs and LPS-G stimulation to obtain more meaningful data.

Human GMSCs are resident in the gingival connective tissues, showing the ability to maintain, repair, and remodel the extracellular matrix for tissue homeostasis [33]. Several studies reported that hGMSCs showed the features and functions of mesenchymal stem cells, such as proliferation, migration and cell differentiation capacity. As demonstrated by our obtained results, the hGMSCs can be considered as MSCs as they follow Dominici’s criteria: they showed the capacity to adhere to a plastic substrate, the fibroblast-like morphology, the capacity to differentiate into adipogenic and osteogenic lineages, positivity to CD73, CD90 and CD105 and negativity for CD14, CD34 and CD45.

The effect of LPS-G stimulation may induce an inflammatory response in hGMSCs that could reproduce similarly the periodontitis microenvironment [34]. The most studied complex involved in intracellular inflammation is the NLRP3 inflammasome, which can be activated when cells are exposed to a wide range of bacterial ligands, including LPS-G [35]. The NLRP3 inflammasome contains a sensor molecule, which can initiate the inflammatory responses [36]. Both the NLRP3 inflammasome and TLR4 signaling play a key role in tissue injury. TLR4 acts as a fundamental factor that promotes the inflammatory response chain in the body [37]. The inhibition of or decrease in the NLRP3/TLR4 signaling pathway is a promising potential target in several chronic diseases [38,39]. Indeed, TLR4 activation triggers the myeloid differentiation factor 88 (MyD88) signaling pathway, which initiates the rapid activation of nuclear factor-kB (NFκB), which leads to an increase in IL-18, IL-6, IL-1β, tumor necrosis factor-α (TNF-α) and monocyte chemotactic protein-1 (MCP-1) [40,41]. Caspase-1 is considered another inflammasome component with a specific role in periodontal disease. Moreover, Caspase-1 was expressed in clinical samples collected from patients affected by periodontal disease.

During periodontal disease progression, IL-1β plays a pivotal role in the induction and maintenance of the host immune response, with a negative effect on periodontal soft tissues. IL-1β expression is regulated by the activation of the NLRP3 inflammasome complexes and subsequent modulation of this intracellular pathway may cause a reduction in IL-1β release [42,43]

The current experiments were designed to evaluate the potential positive effect of AA in response to the inflammasome activation of LPS-G stimulated cells. In the present study, an in vitro model was established where hGMSCs were treated with LPS-G to mimic the periodontal microenvironment. The use of endothelial committed hGMSCs could represent a starting point, as an in vitro model, to evaluate the intracellular signaling pathway that may link the periodontal and cardiovascular diseases. The endothelial commitment of hGMSCs was demonstrated through the CD31 expression evaluated by confocal microscopy observation; cells showed positivity for CD31 at the cytoplasmic level. Moreover, the e-hGMSCs showed the ability to form a capillary-like tube network in a plate coated with Cultrex, indicating that they were committed towards an endothelial phenotype [44]. In fact, tubule formation is one of the hallmarks of angiogenesis, along with cell proliferation and migration [45].

AA (or vitamin C) is a hydrophilic vitamin with high activity as an antioxidant [46]. AA showed the capacity to downregulate ROS production and exert a positive regulatory effect to maintain periodontal health [47]. Moreover, previous findings evidenced that the risk of CVD is correlated inversely with vitamin C diet supplementation [48,49]. The molecular mechanisms involved in the antioxidant property of AA to prevent and to treat the cardiovascular disorders remain elusive [50,51]. Earlier studies have investigated the signaling pathways involved in the effects of AA on CVD related to periodontal disease [52].

In our in vitro model, we determined that stimulation with LPS-G showed a reduction in cell metabolic activity and upregulation of TLR4, MyD88, NFκB, NLRP3, Caspase-1 and IL-1β in hGMSCs and in e-hGMSCs compared to the untreated cells, as demonstrated by immunofluorescence detection and protein level quantization. Indeed, the co-treatment of LPS-G and AA showed a downregulation of TLR4, MyD88, NFκB, NLRP3, Caspase-1 and IL-1β, as demonstrated by qualitative and quantitative analyses, when compared to the cells stimulated with LPS-G alone. Accordingly, the modulation of TLR4 signaling and the NLRP3 inflammasome may provide a novel and valid approach to investigate, in vitro, the progression of the inflammatory cascade in cells exposed to LPS-G stimulation.

In agreement with previous studies [53,54,55], the data obtained in the present work demonstrated that treatment with AA showed a reduction in the expression levels of the NLP3 inflammasome that led to a reduction in the production of IL-1β through the TLR4/MyD88/NFκB signaling pathway in the hGMSCs and e-hGMSCs. Moreover, our findings evidenced a protective role played by AA through the modulation of TLR4/MyD88/NFκB/NLRP3/Caspase-1/IL-1β, reestablishing the microenvironment before LPS-G stimulation.

Furthermore, in the same in vitro model, we investigated ROS production in the same experimental groups. Several studies have demonstrated that ROS production and NLRP3 inflammasome activation are stimulated by various pathogens and metabolic stresses [56]. A high level of ROS induced the activation of the NLRP3 inflammasome in the livers of fructose-fed rats and also enhanced the secretion of IL-1β and IL-18 in rat hepatocytes [56,57]. This study showed that in cells treated with ascorbic acid, high production of ROS is present in hGMSCS and e-hGMSCs stimulated with LPS-G, while the ROS levels are reduced in cells co-treated with AA and LPS-G. Untreated and AA-treated cells showed no differences in ROS production. In fact, as reported by Jung-Yoon Choe et al., AA showed a protective effect against ROS formation and consequent activation of the NLRP3 inflammasome in human macrophages [58].

Although the present study possessed some limitations due to the use of an in vitro model considering one inflammation factor, it may provide a starting point for the evaluation of one of the intracellular mechanisms that occurs during periodontitis. Further studies are required to verify and expand our knowledge on the role of AA in the regulation of the protein and genetic factors that are involved in periodontal disease.

## 5. Limitations of the Study

Although the current in vitro study has provided important information on basic intracellular mechanisms that occur during periodontal disease, it is still far from addressing specific experimental questions presented in the in vivo system. Moreover, to better evaluate the specific role of AA in terms of the neutralization of LPS-G effects or the combination with cell epitopes blocking access to TLR4, further studies are needed to better clarify the molecular mechanisms that are the basis of the use of multiple products. Furthermore, this study was conducted in vitro, and the findings need to be evaluated also through in vivo assays.

## 6. Conclusions

In summary, the present study demonstrated the protective effects of AA through the modulation of TLR4/MyD88/NFκB/NLRP3/Caspase-1/IL-1β in an in vitro model of LPS-G-stimulated cells. These findings suggest the use of AA as a promising potential factor for the prevention of the triggering of the inflammatory cascade that may lead to periodontal and vascular damage. Further studies are necessary to better investigate the molecular mechanisms of these pathways and their role in the progression of periodontal disease.

## Data Availability

Not applicable.
